# Feasibility and Acceptability of Technology-Based Exercise and Posture Training in Older Adults With Age-Related Hyperkyphosis: Pre-Post Study

**DOI:** 10.2196/12199

**Published:** 2019-01-21

**Authors:** Wendy B Katzman, Amy Gladin, Nancy E Lane, Shirley Wong, Felix Liu, Chengshi Jin, Yoshimi Fukuoka

**Affiliations:** 1 Department of Physical Therapy and Rehabilitation Science University of California, San Francisco San Francisco, CA United States; 2 Department of Physical Therapy Kaiser Permanente San Francisco Medical Center Kaiser Permanente Northern California San Francisco, CA United States; 3 Center for Musculoskeletal Health School of Health University of California, Davis Davis, CA United States; 4 Department of Epidemiology and Biostatistics University of California, San Francisco San Francisco, CA United States; 5 Department of Physiological Nursing University of California, San Francisco San Francisco, CA United States

**Keywords:** acceptability, exercise, feasibility, hyperkyphosis, kyphosis, posture, spine, technology-based

## Abstract

**Background:**

Hyperkyphosis is common among older adults and is associated with multiple adverse health outcomes. Kyphosis-specific exercise and posture training programs improve hyperkyphosis, but in-person programs are expensive to implement and maintain over long periods. It is unclear if a technology-based posture training program disseminated through a mobile phone is a feasible or acceptable alternative to in-person training among older adults with hyperkyphosis.

**Objective:**

The primary purpose was to assess the feasibility of subject recruitment, short-term retention and adherence, and acceptability of a technology-based exercise and posture training program disseminated as video clip links and text messaging prompts via a mobile phone. The secondary purpose was to explore the potential efficacy of this program for kyphosis, physical function, and health-related quality of life in older adults with hyperkyphosis.

**Methods:**

In this 6-week pre-post design pilot trial, we recruited community-dwelling adults aged ≥65 years with hyperkyphosis ≥40° (±5°) and access to a mobile phone. The intervention had two parts: (1) exercise and posture training via video clips sent to participants daily via text messaging, including 6 weekly video clip links to be viewed on the participant’s mobile phone, and (2) text messaging prompts to practice good posture. We analyzed the subject recruitment, adherence, retention, and acceptability of the intervention. Outcomes included change in kyphometer-measured kyphosis, occiput-to-wall (OTW) distance, Short Physical Performance Battery score, Scoliosis Research Society (SRS-30) score, Center for Epidemiological Studies Depression score, and Physical Activity Scale for the Elderly (PASE) score.

**Results:**

A total of 64 potential participants were recruited, 17 were enrolled, and 12 completed postintervention testing at 6 weeks. The average age was 71.6 (SD 4.9) years, and 50% were women. The median adherence to daily video viewing was 100% (range, 14%-100%) and to practicing good posture at least three times per day was 71% (range, 0%-100%). Qualitative evaluation of intervention acceptability revealed that the mobile phone screen was too small for participants to view the videos well and daily prompts to practice posture were too frequent. Kyphosis, OTW distance, and physical activity significantly improved after the 6-week intervention. Kyphosis decreased by 8° (95% CI –12 to –5; *P*<.001), OTW decreased by 1.9 cm (95% CI –3.3 to –0.7; *P*=.007), and physical activity measured by PASE increased by 29 points (95% CI 3 to 54; *P*=.03). The health-related quality of life SRS-30 score increased by 0.11 point (SD 0.19), but this increase was not statistically significant (*P*=.09).

**Conclusions:**

Technology-based exercise and posture training using video clip viewing and text messaging reminders is feasible and acceptable for a small cohort of older adults with hyperkyphosis. Technology-based exercise and posture training warrants further study as a potential self-management program for age-related hyperkyphosis, which may be more easily disseminated than in-person training.

## Introduction

Interventions that reduce or slow the progression of age-related hyperkyphosis could have a positive impact on the health status among aging populations. A thoracic spine curvature greater than 40° is commonly defined as hyperkyphosis [1,2] and is prevalent among up to 40% of older adults [3,4]. Kyphosis progresses with age [3,2,5,6], and age-related hyperkyphosis is associated with reduced health-related quality of life (HRQoL), impaired physical function, falls, and elevated fracture risk, particularly when kyphosis progresses to ≥53° [7-13,4]. Recent randomized controlled trials demonstrated improvement in kyphosis and HRQoL after in-person physical therapist-guided kyphosis-specific spine-strengthening exercise and postural training programs over 3-6 months conducted in small groups of older adults with hyperkyphosis [14,15]. However, these labor-intensive programs are expensive to implement and maintain over longer periods of time in clinical and community settings.

One way to reduce the costs of such programs is to utilize digital technologies such as mobile phones, mobile apps, and text messages [16], which are becoming popular communication channels for older adults [17]. However, it is not known whether a technology-based kyphosis exercise and posture training program could provide an alternative self-management intervention that is acceptable and more easily disseminated than in-person training. According to a recent systematic review [18], several factors influence acceptance of technological devices that enhance aging in place for community-dwelling older adults. Older community-dwelling adults have concerns about the high cost, privacy issues, and usability of technological devices. They often question the perceived usefulness and need for technology over more traditional alternatives such as joining a local fitness center for an individual or a group-led exercise program. Older adults are concerned about the social influence of technology use and often look to friends or family for approval or recommendations of new technology. Nevertheless, there has been a rapid rise in mobile phone use among older populations over the past decade and in internet-based self-management programs [19] aimed at increasing physical activity and improving other chronic medical conditions in older adults. Studies suggest that older participants could easily handle the technology after initial training [16,20,21] and, in fact, are more likely to adhere to technology-based interventions [20,22,23]. A technology-based kyphosis exercise and posture training program, disseminated through a mobile phone, may be an acceptable alternative to in-person training for older adults with hyperkyphosis, but the feasibility and acceptability of this type of technology-based program have not been tested thus far.

The primary purpose of this study was to assess the feasibility of subject recruitment, retention and adherence, and acceptability of a technology-based exercise and posture training program disseminated as video clip links and text messaging prompts via a mobile phone. The secondary purpose was to explore the potential efficacy of this program on kyphosis, HRQoL, and physical function in older adults with hyperkyphosis.

## Methods

### Study Design and Participants

This study was a 6-week pre-post pilot trial. The study protocol was approved by the University of California, San Francisco (UCSF), Institutional Review Board prior to participant recruitment and enrollment, and all participants signed an informed consent. Participants were recruited from community talks, university research databases of older adults who had previously agreed to be contacted for future studies, and dental and physical therapy clinics at the UCSF Medical Center. Eligibility criteria were age ≥65 years, kyphometer-derived kyphosis measurement ≥40° (±5°), ability to walk ≥1 block independently without an assistive device, and access to a mobile phone. Participants were excluded for cognitive impairment (inability to draw a normal clock or recall any words on the Mini-Cog [24]). We recruited a small sample to determine feasibility and acceptability and provide preliminary estimates of the effects and SD in clinical measures of kyphosis, HRQoL, and physical function in older community-dwelling adults with hyperkyphosis.

### Screening/Baseline Visit

Interested participants were initially screened by telephone, and those who met the preliminary inclusion criteria were scheduled for a face-to-face screening/baseline visit where baseline kyphosis was measured. Participants meeting all eligibility criteria were enrolled in the study and later attended a 30-minute face-to-face session (intervention) with the study coordinator who provided an overview of the 6-week program and a study manual that coincided with the study video clips including pictures and instructions for exercise and good posture during activities of daily living. This session included two components: instructions and practice in the technical aspect of logging in to the university and library websites to view the video clips and instructions and practice in responding to the text messaging prompts. Each participant was given a unique password and log-in instructions to the university and library websites.

### Intervention

#### Design

The intervention was a 6-week exercise and posture training program comprising two parts: (1) exercise and posture training sent to participants on a daily basis via text messaging, which included a weekly video clip link to be viewed on the participant’s mobile phone, and (2) text messaging prompts to practice good posture. Participants were instructed to view the weekly video clip once per day and practice good posture at least three times per day. The content of the intervention was tested in our previous randomized controlled trials [25,15]. The length of the intervention was chosen because it takes a minimum of 2 weeks to master learning, and a minimum of 6 weeks to adapt to exercise [26].

#### Exercise and Posture Training via Video Clips

**Figure 1:**
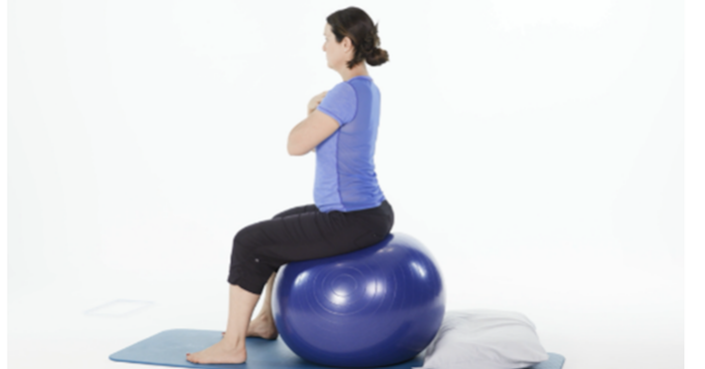
Screenshot of the exercise video on neutral pelvic alignment.

The video clips were 45- to 60-second demonstrations of 6 lessons in exercise and posture training that taught participants good postural alignment and movement during activities of daily living (Table 1). Participants received daily text messages with a link to the specific weekly video lesson. The person in the video clip demonstrated ideal spinal alignment during activities of daily living, serving as a role model for good posture (Figure 1), and the pictures in the training manual reinforced the weekly lesson. We established a link to the study on the university library website, where participants could view the 6 video clips. Once the study coordinator enrolled participants in the study, they were able to view the study videos after successfully logging in to the study site on the library website. The log-in process involved 2 steps: log in to the university website with a unique password and then log in to the library website using the same password. The library website collected analytics that quantified viewing time for each participant for each video.

#### Text Messaging Prompts

Participants received daily reminders to practice good posture at least three times a day during their daily activities. At the onset of the study, participants could choose the frequency of text messaging reminders and whether they wanted to receive one, two, or three daily reminders. All participants were texted at the end of the day and instructed to reply to the question, “Did you practice at least 3 times today?” Participants were prompted to reply by text with 1 (yes) or 0 (no) every day during the 6-week program.

Text messages were sent automatically via the company, Twilio [27], a cloud communications platform service specifically programmed for this study. This tool allowed the research team to schedule the text messages for every participant and monitor and confirm delivery and individual responses.

**Table 1:** Exercise and posture training intervention: Weekly video lessons and training activities.

Week	Video lessons and training activity
1	Practice good standing posture
2	Practice good sitting posture
3	Bend from the hips and knees, and keep the spine straight
4	Neutral pelvic alignment supports good posture
5	Build spine strength with exercise
6	Improve balance

#### Postintervention Visit

After completing the 6-week pilot study, all outcome measures were repeated, and participants were interviewed by the study coordinator using a semistructured interview guide designed to explore participants’ experiences and perspectives on acceptability of using the mobile phone and video-clip technology.

### Measurements

#### Feasibility of Recruitment, Retention, and Adherence

We determined the number of participants who completed the telephone screening, how they heard about the study recruitment, the number of participants who met the eligibility criteria, and the number who completed the 6-week study visit. At baseline, we assessed self-efficacy of adherence to the intervention and asked participants how confident they were that they would practice good posture at least three times a day, watch the video daily, and reply to a text daily during the 6-week intervention.

#### Adherence to Video Viewing

The duration of viewing time for each video was recorded for every participant using the university library website analytics. Weekly adherence to video viewing was calculated as a percentage of the actual duration of viewing time/the expected duration of daily viewing time each week × 100. Maximum possible adherence was 100%, even if participants exceeded the expected viewing time.

#### Adherence to Practicing Good Posture at Least Three Times a Day

A text message question was sent to each participant at the end of each day, which asked them whether they practiced the exercise at least three times that day, and their responses were recorded in the study database. Weekly adherence to practice was calculated as a percentage of the number of actual days of practice at least three times divided by the expected days of practice at least three times × 100.

#### Qualitative Exploration of the Pilot Study

The study coordinator asked participants the following 7 questions at the postintervention visit: (1) Looking back over the last 6 weeks, what did you learn the most from the study? (2) What did you like the most about the study? (3) What did you like the least about the study? (4) What would you change about the study? (5) What do you feel would have motivated you more to improve your posture? (6) What advice would you give to other older adults to help improve their posture? (7) Is there anything else you would like to add? The responses were recorded and summarized.

#### Kyphosis, Physical Function, and Other Outcomes

Kyphosis and physical function measurements were performed at a university-based physical performance laboratory by a trained exercise physiologist before and after the 6-week intervention. The remaining questionnaires were administered by the study coordinator, and participants completed the questionnaires on a study iPad (Apple Inc, Cupertino, CA).

##### Kyphosis

**Figure 2:**
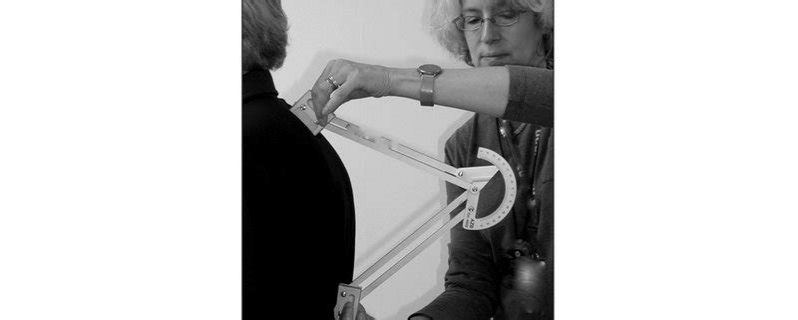
Kyphometer measurement of kyphosis.

**Figure 3:**
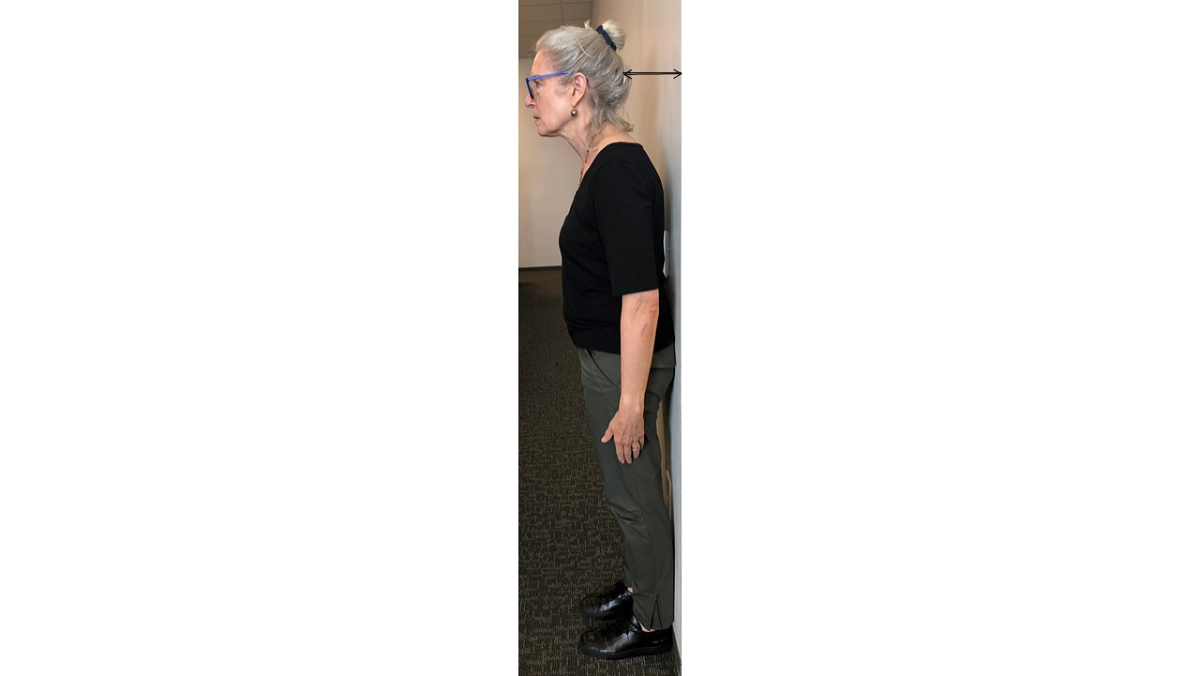
Measurement of occiput-to-wall distance.

Kyphosis was measured in degrees using a standardized protocol for clinically measured kyphosis (T3-T12) [28] and the Debrunner kyphometer while the participant stood in his/her usual posture (Figure 2). A higher degree of kyphosis indicated worse kyphosis [29-31]. The intraclass correlation coefficient for repeated observer analysis of kyphosis measurements using the Debrunner kyphometer is 0.95 [32]. Occiput-to-wall (OTW) distance, a surrogate clinical measure of kyphosis [33,34], was acquired with two rulers placed perpendicular, one vertically behind the head at the occiput and one horizontally measuring the distance from the wall to the vertically placed ruler, while the participant stood with both heels and the sacrum against the wall with the head positioned in the Frankfort horizontal plane (Figure 3). OTW distance >5 cm predicts a risk of hyperkyphosis [33,35]. Reproducibility of the OTW distance among older adults aged >60 years with excessive kyphosis was 0.99 for intrarater reliability and 0.93 for interrater reliability [36].

##### Physical Function

The Short Physical Performance Battery (SPPB) consists of three areas of lower-extremity function including static balance, gait speed, and getting in and out of a chair. Each area is scored individually (0-4 points) with a composite SPPB score of 0-12 points; higher scores indicate better physical performance [37].

##### Physical Activity

The Physical Activity Scale for the Elderly (PASE) questionnaire is a brief survey designed specifically to assess frequency, duration, and intensity level of physical activity over the previous week in persons aged ≥65 years. Scores range from 0 to 793 points, with higher scores indicating greater physical activity [38].

##### Depression Symptoms

The Center for Epidemiological Studies Depression (CESD) is a self-reported depression symptom scale. The possible range of scores is 0-60 points, with higher scores indicating more symptomatology and scores ≥16 indicating the presence of depression [39,40].

##### Health-Related Quality of Life

The modified Scoliosis Research Society (SRS-30) is a self-reported spine-specific quality-of-life instrument that includes four domains scored separately, with an overall mean composite score of 1-5 points (1=worst, 5=best). Domains include function/activity, pain, mental health, and self-image/appearance and an additional score for satisfaction with management [41].

### Statistical Analyses

Baseline demographic characteristics of the enrolled participants were summarized using mean, SD, and range for continuous measures. Tabulations and percentages were used to summarize categorical variables. We used descriptive statistics of mean with SD and median to characterize process measures required to demonstrate adherence to the intervention. Kyphosis, physical function, and HRQoL scores at baseline and 6 weeks postintervention were summarized with means and SDs. Paired *t* tests were used to assess the effects of the intervention on changes in the scores from baseline to the end of the 6-week intervention period. The results were also reported with confidence intervals and *P* values. All analyses were conducted using SAS software, version 9.4 (SAS Institute Inc, Cary, NC).

## Results

### Feasibility of Recruitment, Retention, and Adherence

**Figure 4:**
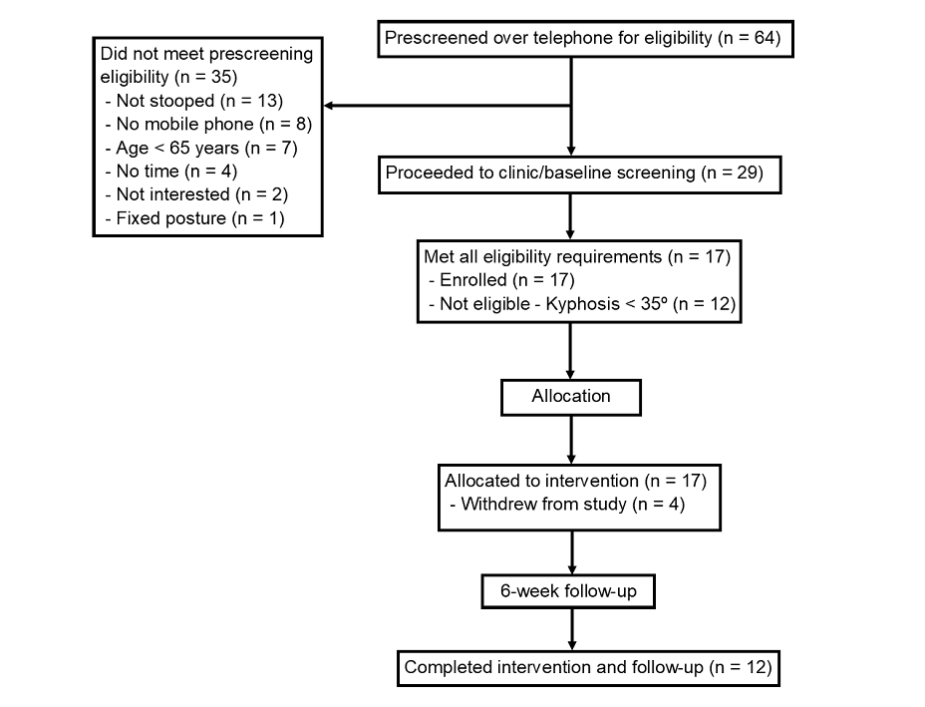
Participant recruitment and retention.

A total of 64 potential participants were recruited between January and May 2018, of which 29 met the preliminary eligibility criteria by telephone and were scheduled for an in-person clinic/baseline visit. Twelve potential participants did not meet the kyphosis eligibility criteria (kyphosis ≤40° ± 5°), and 17 (58.6%) met all eligibility criteria and were enrolled in the study (Figure 4). The 12 participants who completed the intervention and follow-up visit had a mean age of 71.6 (SD 4.9) years, and 92% had college, professional, or graduate degrees (Table 2). As per the baseline self-efficacy scales, participants were 97% confident they would practice good posture at least three times a day, 97% were confident that they would watch a daily video, and 98% were confident that they would reply to a daily text. Five participants did not complete the study. One dropped out for medical reasons, two dropped out during the first 2 weeks due to frustration with the two-step log-in process, one lost interest after 2 weeks, and one completed all aspects of the intervention but did not return for the 6-week postintervention testing. The mean age of the 5 participants (two male, three female) who did not complete the 6-week follow-up visit was 71.5 years, and 80% had college, professional, or graduate degrees (one unknown). The one participant who completed the intervention but did not return for the 6-week follow-up visit reported 50% confidence on the self-efficacy scales at baseline.

#### Adherence to Video Viewing

The mean adherence to video viewing over the 6-week study (n=12) ranged from 76% to 87% among those who completed the 6-week testing visit (Table 3). The median adherence to video viewing was 100% (range, 14%-100%).

#### Adherence to Practicing Good Posture at Least Three Times a Day

The mean adherence to practicing at least three times a day over the 6-week study ranged from 62% to 77% among those who completed the 6-week testing visit (n=12) (Table 3). The median adherence to practice was 71% (range, 0-100).

#### Preliminary Estimates of Change in Outcome Measures

Kyphosis, OTW distance, and physical activity measured by the PASE questionnaire significantly improved after the 6-week intervention (Table 4). Kyphosis decreased by 8° (95% CI –12 to –5; *P*<.001), the OTW distance decreased by 1.9 cm (95% CI –3.3 to –0.7; *P*=.007), and the physical activity score increased by 29 points (95% CI 3 to 54; *P*=.03). The SRS-30 quality-of-life composite score increased by 0.11 point (SD 0.19; *P*=.09), and individual self-image/appearance, pain, and function/activity domain scores were higher after the intervention, but not statistically significant (*P*>.05; data not shown).

#### Qualitative Exploration of the Pilot Study

Results from the semistructured interviews completed at the 6-week postintervention visit are summarized in Table 5. Participants had difficulty with the video technology interface, difficulty watching the videos on their phones, and questions about dosing of the posture instructions received in the videos and found the daily texts bothersome. Participants reported preferences for less frequent text messaging, ability to view the videos on a larger screen, more clarity regarding time spent practicing, and an easily accessible platform for viewing the videos.

**Table 2:** Demographic characteristics of enrolled participants at baseline (N=12).

Variable		All participants
Age (years), mean (SD), range		71.6 (4.9), 65-81
**Self-efficacy scale^a^, mean (SD), range**		
	Practice good posture at least three times a day	9.7 (0.65), 8-10
	Watch a daily video	9.7 (0.65), 8-10
	Reply to a daily text	9.8 (0.45), 9-10
Sex (female), n (%)		6 (50)
Race/ethnicity (Caucasian), n (%)		11 (92)
**Education, n (%)**		
	Some college, vocational, or high school	1 (8)
	College graduate	5 (42)
	Professional or graduate degree	6 (50)
Paid part-time or full-time job (yes), n (%)		4 (33)
**Co-morbidities, n (%)**		
	0-1	7 (58)
	≥2	5 (42)
**Type of mobile phone, n (%)**		
	Android	3 (25)
	iPhone	9 (75)

^a^Score ranges from 0 to 10 points and a higher score indicates a higher self-efficacy.

**Table 3:** Adherence to daily video viewing and practicing over the 6-week pilot study.

Activity	Adherence, mean (SD)						Adherence, median (range)
	Week 1	Week 2	Week 3	Week 4	Week 5	Week 6	Weeks 1-6
Watched video daily^a^	76 (29)	87 (28)	84 (25)	79 (33)	76 (32)	76 (29)	100 (14-100)
Practiced at least three times daily^b^	62 (36)	75 (24)	77 (13)	77 (21)	64 (27)	70 (26)	71 (0-100)

^a^Adherence to video viewing calculated as actual viewing time divided by the total possible viewing time × 100 (maximum adherence was reported as 100%).

^b^Adherence to practice calculated as the number of actual days of practice at least three times divided by total possible number of days practiced at least 3 times × 100. Missing data on one participant who practiced the exercise, but failed to reply to daily texts.

**Table 4:** Baseline, postintervention, and preliminary estimates of change in outcome measures for participants who completed the 6-week testing visit.

Outcome measure	Baseline, mean (SD)	Postintervention, mean (SD)	Change^a^, mean (SD)	*P* value for change	95% CI for change
Kyphosis degree derived using kyphometer^b^ (degrees)	51 (10)	43 (12)	-8 (5)	<0.001	–12 to –5
Occiput-to-wall distance^b^ (cm)	7.8 (4.1)	5.9 (3.2)	–1.9 (2.1)	0.007	–3.3 to –0.7
Short Physical Performance Battery (0-12 points)	11.1 (1.0)	10.9 (1.3)	–0.2 (1.0)	0.59	–0.8 to 0.5
Scoliosis Research Society (0-5 points)	3.94 (0.23)	4.05 (0.25)	0.11 (0.19)	0.09	–0.02 to 0.23
Physical Activity Scale for the Elderly (0-793 points)	109 (68)	138 (55)	29 (40)	0.03	3 to 54
Center for Epidemiological Studies Depression score^b^ (0-60 points)	6.6 (4.9)	6.1 (3.7)	–0.4 (3.8)	0.71	–3 to 2

^a^Change reported for participants with baseline and 6-week post-intervention scores (n=12).

^b^Higher scores indicate worse kyphosis, occiput-to-wall distance, and CESD; negative change indicates improvement.

**Table 5:** Content of intervention, results, and lessons learned from the 6-week pilot study.

Content of intervention	Results	Lessons learned
Text messaging reminders sent to participants to practice good posture one, two, or three times a day every day for 6 weeks (no response requested)	Participant requests for text messaging reminders varied from one to three times a day	Daily text reminders too frequent for most participants (n=5)
Instructions to reply to the question, “Did you practice at least 3 times today”? (yes/no) every day for 6 weeks	Adherence to practicing at least three times a day: median (range)=71% (0%-100%)	Good adherence to practice at least three times a day
Instructions to watch video clips daily on a UCSF^a^ library site (remote intervention)	Adherence to watching the videos declined after the second week from 87% to 76%; median (range) adherence=100% (14%-100%)	Cell phone screen too small to view videos (n=3); two-step log-in process was cumbersome (n=8)

^a^UCSF: University of California, San Francisco.

## Discussion

### Principal Results

We explored the feasibility of subject recruitment, retention, and acceptability of an exercise and posture training program sent as video clip links and text messaging prompts via a mobile phone to older adults with hyperkyphosis. Only 8 (12.5%) participants who were screened for the study did not qualify due to no access to a mobile phone, highlighting prior reports that technology use is rapidly increasing in older populations [17]. Of those who did not complete the 6-week postintervention visit, two dropped out during the first 2 weeks because of frustration with the two-step log-in procedures; we learned that a two-step process is too cumbersome, even for those who completed the study. Overall, participant acceptance and satisfaction with the intervention was positive. Adherence to the intervention was high among those who completed the 6-week intervention. Based on participant feedback, we learned that daily text messaging reminders were too frequent and participant preference for frequency of text messaging reminders is an important consideration for future studies. Furthermore, the mobile phone screen was too small for easy viewing, and future modifications should include access to the video for viewing on a larger computer screen and an accessible log-in without the two-step process. To investigate the effects of a technology-based intervention on kyphosis and kyphosis progression in the future, we plan to conduct a longer intervention with regular interactive video webinars to keep participants engaged, and provide online demonstrations and feedback to participants. These webinars could be accessed from individual personal computers that provide a larger screen and avoid the cumbersome two-step log-in process that our participants found difficult.

Kyphosis decreased by 8° (95% CI –12 to –5), OTW distance decreased by 1.9 cm (95% CI –3.3 to –0.7), and PASE score increased by 29 points (95% CI 3 to 54), which indicates improvement in clinical measures of kyphosis and physical activity, although we did not have a large enough sample to assess the potential efficacy of this program on progression of kyphosis, OTW distance, and physical activity in older adults with hyperkyphosis. Results of this study suggest that delivering an exercise and posture self-management program via technology is promising and deserves further investigation.

### Comparison with Prior Work

Our results are consistent with studies showing that self-management programs that focus on day-to-day management of chronic diseases significantly improve heath behaviors and health status [42,43]. At baseline, our participants reported a high likelihood of adherence to the proposed self-management intervention, an indication of high self-efficacy that has been associated with a significantly higher likelihood of good outcomes among older adults with knee osteoarthritis [44,45]. High self-efficacy has also been shown to predict successful integration of other healthy behaviors and enhance sustainability of high levels of self-care [46,47].

Results of our study are also consistent with those of a previous systematic review that reported that text messaging reminders enhanced participants’ abilities to self-manage their chronic condition (asthma, diabetes, or hypertension) [48]. Text messaging reminders have demonstrated efficacy in improving adherence to a variety of health behaviors across multiple domains [49-51]. Moreover, participants who perceived improvements in self-management preferred reminders via mobile phone messaging over email reminders, highlighting the acceptance and usability of mobile phone technology in older populations [48]. Another systematic review of smart technology interventions aimed at facilitating, supporting, and sustaining self-management through behavioral change in people with chronic obstructive pulmonary disease, concluded that the use of technology improved HRQoL and physical activity compared to face-to-face or digital/written support [52] and continued use of a smart technology intervention improves sustainability of behavior change over time.

The improvement in kyphosis in our study exceeds that reported in previous randomized controlled trials that tested the efficacy of in-person exercise and posture training interventions in older adults with hyperkyphosis over longer periods of time [14,15]. We used trained testers at the University’s Clinical and Translational Science Institute’s physical performance laboratory, provided additional training for our outcome measurements, and ensured high within- and between-tester reliability prior to the study. Although there were large SDs in the kyphosis measurements and a very small sample size, the change in kyphosis exceeded the standard error of the measurement. It is possible that our technology-based educational intervention improved motor control and provided participants self-management tools and greater autonomy for improving posture as compared to prior in-person interventions.

There are no prior studies that specifically target OTW distance; however, the 1.9-cm change in OTW distance observed in our study did not exceed the smallest detectable difference of 3.2 cm according to Bland-Altman criteria previously reported among adults with ankylosing spondylitis [34]. In contrast, the PASE scores of physical activity improved by 29 (SD 40) points, which is a robust 27% improvement from the baseline mean score of 107 (SD 61) points to 138 (SD 55) points at the 6-week visit. The mean score for older adults aged ≥65 years was 102.9 (SD 64.1) points, suggesting that educating participants about their posture may also increase their participation in physical activities compared to their age-matched peers [38]. Participants reported greater frequency and duration of walking and performing more yard work and home repair after the intervention. It is possible, although unlikely, that this additional physical activity contributed to a change in kyphosis, but we cannot discount the possibility that the skills learned from the intervention are responsible for both the increase in physical activity and improved kyphosis. 

### Limitations

This pilot study may be the first of its kind to investigate the feasibility of delivering a technology-based kyphosis-specific exercise and posture-training program by mobile phone over 6 weeks in older community-dwelling adults with hyperkyphosis. These programs have previously required in-person training for 3-6 months. However, there are several limitations. First, this was a pilot study without a control group, and the results may be larger than a between-group comparison in a controlled trial. Second, we excluded participants who were not English speakers and did not have access to a mobile phone, tablet, or computer. In addition, most of our participants were white and had college degrees or graduate degrees, which limits our ability to generalize the preliminary results to the overall older adult population with hyperkyphosis. Third, our sample size was small with large SDs in the effects; however, these data will be helpful in designing a future randomized controlled trial in a large sample. Fourth, failure to complete all aspects of the study was high, although one participant dropped out for medical reasons unrelated to the study, three dropped out within the first 2 weeks, and one completed all aspects of the study except the 6-week testing. This drop-out rate is within the range of 6% to 34% reported in exercise interventions in older persons. In addition, the early drop-out rate observed in our study is consistent with that in previous studies reporting the highest number of dropouts in the first 3 months of longer-term studies [53]. Fifth, our sample was highly educated and implementing this study in a lower-educated population may be challenging. However, a systematic review that described characteristics of community-dwelling older adults that influence acceptance of technology listed the familiarity of older adults with modern technology, the fit between the housing type and technology, and the compatibility of the technology with adults’ cultural background, rather than educational level, as limiting factors [18]. Future randomized studies to determine the efficacy of this technology among larger and more diverse populations are needed.

### Conclusions

Technology-based exercise and posture training using video clip viewing and text messaging reminders is feasible and acceptable in a small cohort of older adults with hyperkyphosis. Adherence to video viewing is excellent and adherence to practicing exercise at least three times a day warrants further study to optimize adherence and the optimal dose. Future trials should assess the benefits of more individualized feedback with posture training videos and customize daily text reminder prompts according to participant preference. Technology-based exercise and posture training in older adults with hyperkyphosis warrants further study as a potential self-management program for age-related hyperkyphosis that may be more easily disseminated than in-person training.

## Abbreviations

CESD: Center for Epidemiological Studies Depression
HRQoL: health-related quality of life
OTW: occiput to wall
PASE: Physical Activity Scale for the Elderly
SPPB: The Short Physical Performance Battery
SRS-30: Scoliosis Research Society
UCSF: University of California, San Francisco
